# Short-Term Sleep Fragmentation Dysregulates Autophagy in a Brain Region-Specific Manner

**DOI:** 10.3390/life11101098

**Published:** 2021-10-16

**Authors:** Yan Cheng, Woong-Ki Kim, Laurie L. Wellman, Larry D. Sanford, Ming-Lei Guo

**Affiliations:** 1 Center for Integrative Neuroscience and Inflammatory Diseases, Eastern Virginia Medical School, Norfolk, VA 23507, USA; chengy@evms.edu (Y.C.); kimw@evms.edu (W.-K.K.); WellmaLL@evms.edu (L.L.W.); SanforLD@evms.edu (L.D.S.); 2 Sleep Research Laboratory, Department of Pathology and Anatomy, Eastern Virginia Medical School, Norfolk, VA 23507, USA; 3 Drug Addiction Laboratory, Department Pathology and Anatomy, Eastern Virginia Medical School, Norfolk, VA 23507, USA; 4 Department of Microbiology and Molecular Cell Biology, Eastern Virginia Medical School, Norfolk, VA 23507, USA

**Keywords:** sleep fragmentation, microglia, neuroinflammation, autophagy, corticotropin releasing factor

## Abstract

In this study, we investigated autophagy, glial activation status, and corticotropin releasing factor (CRF) signaling in the brains of mice after 5 days of sleep fragmentation (SF). Three different brain regions including the striatum, hippocampus, and frontal cortex were selected for examination based on roles in sleep regulation and sensitivity to sleep disruption. For autophagy, we monitored the levels of various autophagic induction markers including beclin1, LC3II, and p62 as well as the levels of lysosomal associated membrane protein 1 and 2 (LAMP1/2) and the transcription factor EB (TFEB) which are critical for lysosome function and autophagy maturation stage. For the status of microglia and astrocytes, we determined the levels of Iba1 and GFAP in these brain regions. We also measured the levels of CRF and its cognate receptors 1 and 2 (CRFR1/2). Our results showed that 5 days of SF dysregulated autophagy in the striatum and hippocampus but not in the frontal cortex. Additionally, 5 days of SF activated microglia in the striatum but not in the hippocampus or frontal cortex. In the striatum, CRFR2 but not CRFR1 was significantly increased in SF-experienced mice. CRF did not alter its mRNA levels in any of the three brain regions assessed. Our findings revealed that autophagy processes are sensitive to short-term SF in a region-specific manner and suggest that autophagy dysregulation may be a primary initiator for brain changes and functional impairments in the context of sleep disturbances and disorders.

## 1. Introduction

Insufficient sleep time and/or poor quality of sleep are prevalent in modern society due to factors including excessive workloads and high social competition. Adequate sleep is critical for proper brain function [1,2] and substantial evidence demonstrates that chronic sleep insufficiency is associated with cognitive decline and psychological problems ranging from mood changes to psychotic symptoms [3,4]. Alzheimer’s disease (AD) and Parkinson’s disease (PD) patients usually exhibit sleep disturbance in the preclinical and early stages of the disease [5] and sleep disruption without proper medication may accelerate the pathogenesis of these neurodegenerative diseases [6]. Epidemiological studies also show that individuals with sleep disorders have a higher incidence of developing neurodegenerative diseases than do individuals with normal sleep [7]. However, the mechanisms by which sleep disturbances exacerbate brain dysfunctions remain largely unknown.

Autophagy is an evolutionarily conserved process which engulfs improperly folded cytosolic proteins or damaged organelles within double-membrane-bound compartments to form autophagosomes which fuse with lysosomes for eventual degradation [8,9]. Basal and constituent levels of autophagy are critical for cellular homeostasis because they eliminate nonfunctional biomolecules and damaged organelles inside cells. Under challenging conditions such as nutrient depletion or temperature and oxidative stress, autophagy can be induced to degrade certain biomolecules to coordinate metabolic and biogenesis processes that help to ensure cell survival [10]. Autophagy is tightly regulated by the sequential expression of autophagy-related (ATG) genes and alterations in the expression levels of various ATG genes are commonly employed as markers of changes in autophagy activity. Beclin1, a phylogenetically conserved protein essential for autophagy induction, interacts with proteins including ATG14 and phosphatidylinositol 3-kinase catalytic subunit type 3 (PIK3C3) to generate the class III phosphatidylinositol 3-kinase (PtdIns3K) that forms an initiation complex. This complex subsequently promotes the recruitment of certain ATG proteins to facilitate the transformation of LC3BI to LC3BII by the addition of a phosphatidylethanolamine (PE) group to the carboxyl terminus of cytosolic LC3BI. Levels of LC3BII are widely recognized and employed as a quantification marker for autophagosome formation [8,9]. Following formation, autophagosomes fuse with lysosomes to form autolysosomes that degrade the contents inside them. In this process, p62 is degraded reflecting the completion of the autophagy process (autophagy maturation). Normal lysosomal function is critical for autophagy completion. In general, levels of lysosomal associated membrane protein 1 and 2 (LAMP 1/2) and transcription factor EB (TFEB) are employed as indices of lysosome biogenesis and function [11,12].

Microglia, the brain residential macrophage, constitute the first defense line against various types of challenges including viral invasion, stroke, neuronal injuries, and abused drugs, etc. [13,14]. The normal interaction between microglia and neurons is pivotal for brain development as well as for adult brain homeostasis. Once stimulated, microglia may undergo morphological changes accompanied by upregulation in various neuroimmune pathways, which ultimately, lead to increased production and release of a plethora of chemokines and cytokines including TNFα, IL6, and IL1β. These inflammatory mediators then act on neighboring neurons and modulate their excitability [15,16]. Low levels of neuroinflammation may help repair and restore neuronal injuries; however, sustained and chronic neuroinflammation can exacerbate existing brain damage and lead to more severe injuries. Enhanced microglial activation and neuroinflammation levels have been consistently found in the postmortem brains of AD and PD patients and have been implicated as being “driving forces” for their pathogenesis [17,18].

There is inherent cross-talk between autophagy and immune responses and autophagy dysregulation is involved in peripheral and central nervous system inflammation in the context of various neural challenges and insults [19,20]. Previous studies have demonstrated that chronic sleep disturbances can activate microglia leading to increased neuroinflammation [21] and that the inhibition on microglial activation was capable of mitigating sleep deprivation-mediated memory and cognitive loss [22,23]. Autophagy is also dysregulated by chronic sleep disturbance [24,25]. However, to our knowledge, the effects of relatively short-term sleep disruption on autophagy and on microglia and astrocytes have not been determined. In this study, we selected three brain regions including the striatum, hippocampus, and frontal cortex for such exploration. The three selected brain regions are also regulated by sleep activity and are vulnerable to sleep disruption. [26]. Also, we checked the status of corticotropin releasing factor (CRF) signaling in these three regions because CRF and CRF receptors 1 and 2 are well-known to be dysregulated by sleep disturbances and to mediate sleep deprivation-induced biological effects [27,28,29]. In this study, we reveal that 5 days of sleep fragmentation (SF) dysregulated autophagy both in the striatum and hippocampus as well as microglial activation in the striatum. CRFR2 was upregulated in the striatum by 5 days of SF. Our results suggest that autophagy is sensitive to short-term sleep disturbance and suggest that autophagy dysregulation may be involved in brain impairments in the context of sleep disturbances and disorders.

## 2. Methods and Materials

### 2.1. Animal

Ten adult male C57BL/6 mice were obtained from Jackson Lab (Bar Harbor, ME, USA). The mice were 10–12 weeks old and weighed 20–25 g upon arrival. They were housed and were kept in a colony room with food and water available ad libitum. The colony room was maintained on a 12:12 light to dark cycle and ambient temperature at 24.0 °C ± 1.5 °C. All procedures were conducted in accordance with the National Institutes of Health Guide for the Care and Use of Experimental Animals and were approved by Eastern Virginia Medical School’s Institutional Animal Care and Use Committee.

### 2.2. Antibodies and Reagents

The following antibodies were used at the indicated concentration in our studies: astrocyte activation marker GFAP (1:5000, ab7260; Abcam (Boston, MA, USA), beclin1 (1:2000, NB500-249), microglial activation marker Iba1 (1:2000, NBP2-19019), LC3B (1:2000, NB100-2220), LAMP2 (1:2000, NB300-591), LAMP1 (1:2000, NB120-19294), p62 (1:2000, H00008878-M01, Novus Biological (Littleton, CO, USA); TFEB (1:2000; cell signaling technology, #4240; (Danvers, MA, USA), CRFR1 (1:1000, Sigma, SAB4500465); CRFR2 (1:1000, SAB4500467, Sigma (St. Louis, MO, USA), β-actin (1:2000, sc-8432, Santa Cruz (Lincoln, NE, USA) or (Sigma; 1:2000, A2066). Second antibodies were purchased from Li-COR company (Lincoln, NE, USA), (1:10, 000) including IRDye^®^ 680RD Donkey anti-Mouse or rabbit IgG; IRDye^®^ 800CW Donkey anti-Mouse or rabbit IgG. 

### 2.3. Sleep Fragmentation (SF) Procedure

SF was performed using commercial, validated devices (Lafayette Instruments, (Lafayette, IN, USA), Sleep Fragmentation Chamber, model 80391) that employ an automated sweeper arm that moves across animal cages to disrupt sleep via tactile stimulation [30,31]. An SF protocol with 2 min between each sweep reportedly produces moderate to severe SF [30,31,32] without significantly reducing overall sleep or significantly impacting sleep macro- or micro-architecture [30,31]. Studies have also reported that this procedure is not associated with measurable increases in stress hormones [30,31], but can impair cognitive performance [31] and can have a negative impact on health including promoting obesity [33] and tumor formation [32]. This SF device and protocol has been routinely used in our work [34,35].

In brief, all animals undergoing SF (n = 5) were placed into the devices 1 day prior to the start of the SF. For SF, sleep was interrupted at 2 min intervals during the 12 h light period (normal sleeping period of mice). During the 12 h dark period, the motorized mechanical sweeper was stopped and mice were free to behave normally. Animals were observed daily to assess their health and to assure proper functioning of the SF device. Sham animals (n = 5) were maintained in their home cages without any interruption. Sleep was fragmented for 5 days. On the next day, all mice were sacrificed and brain tissue was collected.

### 2.4. Western Blots

The brains were separated by using brain slicer into several regions including the striatum and hippocampus. Separated brain tissues were dissolved in RIPA buffers (Thermo Scientific, (Waltham, MA, USA)) and sonicated for 10 s on ice at an amplitude of 70% (Thermo Scientific). The brain homogenates were then incubated at 4 °C for 30 min followed with 12,000 rpm centrifugation for 10 min. The supernatants were taken out and the protein concentrations were calculated through the BCA method. Equal amounts of the proteins were electrophoresed in a sodium dodecyl sulfate-polyacrylamide gel (160 V, 60 min) under reducing conditions followed by transfer to PVDF membranes (180 mA, 90 min). The blots were blocked with 3% nonfat dry milk in Tris-buffered saline (TBST). The Western blots were then incubated with indicated antibodies overnight at 4 °C. The next day, the membranes were washed and incubated with IRDye fluorescent mouse or rabbit second antibody (1: 10,000, LI-COR Biosciences) for one hour at room temperature. After three washes with TBST, the membranes were put into the Odyssey^®^ Imaging System for image development and the intensity of fluorescent band were quantified using Image Studio™ Software. After imaging, the membranes were re-probed by β-actin for normalization.

### 2.5. RNA Extraction, Reverse Transcription, and Quantitative Polymerase Chain Reaction (qPCR)

Total RNA was extracted using Trizol reagent (Invitrogen (Waltham, MA, USA), 15596-018). Briefly, 100 mg brain tissue was directly added to 1 mL Trizol. Brain lysates were briefly sonicated (3–5 s) and incubated for 10 min on ice then aspirated into new 1.5 mL microcentrifuge tubes with 0.2 mL of chloroform added. After extensive vortex, the samples were centrifuged at 10,000 g for 15 min at 4 °C. The upper aqueous phase was transferred to a new tube and 500 µL isopropyl alcohol was added. Samples were then incubated for 10 min and centrifuged again to precipitate total RNA. The total RNA was dissolved in DEPC-treated H_2_O and quantified. Reverse transcription reactions were performed using a Verso cDNA kit (Invitrogen, AB-1453/B). The reaction system (20 µL) included 4 µL 5X cDNA synthesis buffer, 2 µL dNTP mix, 1 µL RNA primer, 1 µL RT enhancer, 1 µL Verso enzyme Mix (Invitrogen, AB-1453/B), total RNA template 1 µg, and a variable volume of water. Reaction conditions were set at 42 °C for 30 min. Quantitative polymerase chain reactions (qPCRs) were performed by using SuperScript™ III Platinum™ One-Step qRT-PCR Kit (Invitrogen, 11732020). Reaction systems were set up as follows: 10 µL Master mix, 1.0 µL primers and probes, and 1 µL cDNA and 8 µL distilled H_2_O. We placed 96-well plates into a QS3 qPCR machine (invitrogen) for program running. Mouse primers for GADPH and CRF were purchased from (Invitrogen, Mm99999915 and Mm01293920) with GADPH serving as the internal control for quantification.

### 2.6. Statistical Analysis

The results were presented as means ± SEM. For comparisons between two groups an unpaired two-tailed Student’s *t*-test was used; all statistical tests were performed with GraphPad Prism (La Jolla, CA, USA). Probability levels of <0.05 were considered statistically significant. A minimum of four biological replicates were used for all experiments.

## 3. Results

### 3.1. Short-Term SF Dysregulated Autophagy in the Striatum

**Figure 1:**
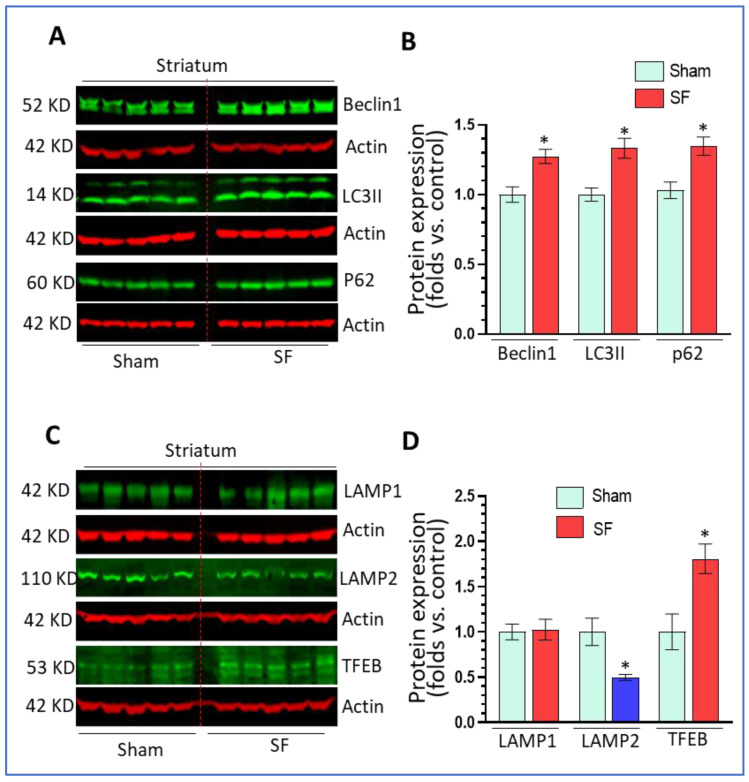
Short-term sleep fragmentation (SF) dysregulated autophagy in the striatum. Mice experiencing 5-day SF or sham controls were sacrificed for brain tissue collection. The striatum was separated for protein (**A**,**B**) Beclin1, LC3II, and p62 significantly increased their levels in the striatum of SF-experienced mouse brains compared to the sham controls (* *p* < 0.05, n = 5). (**C**,**D**) Lysosomal associated membrane protein 2 (LAMP2) and transcription factor EB (TFEB) significantly decreased and increased their levels, respectively, in the striatum of SF-experienced mouse brains compared to the sham controls (* *p* < 0.05, n = 5). Lysosomal associated membrane protein 1 (LAMP1) did not show significant changes in its levels between these two groups (* *p* > 0.05, n = 5). For all Western blots, β-actin were served as a protein load control. Data were expressed as means ± SEM and were analyzed using Student’s *t*-tests (* *p* < 0.05 versus sham). (Original Western Blot Figure see Supplementary Figure S1).

Previous investigations demonstrated that long-term SF (two months) dysregulated autophagy in multiple brain regions [36]. Here, we explored whether short-term SF also had effects on autophagy in the brain. After 5 days of SF, the mice showed normal behaviors (eating, drinking, and locomotion) without weight loss compared to sham controls (data not shown). Both groups of mice were sacrificed 1 day after the last session of SF for brain tissue collection. The striatum was separated out for the detection of various autophagic markers including beclin1 (autophagy initiation marker), LC3II (autophagosome marker), and p62 (autophagy flux marker). We selected the striatum because it has been implicated in sleep regulation (reviewed in [37]) and shown sensitive to sleep disruption [26] but has received less attention compared to other regions such as the hippocampus and frontal cortex. The results showed that 5 days of SF significantly increased beclin1 levels (1.27 ± 0.05 fold, *p* < 0.05), LC3II levels (1.33 ± 0.07 fold, *p* < 0.05), and p62 levels (1.35 ± 0.06 fold, *p* < 0.05) (Figure 1A,B) compared to sham. These findings indicate that SF increased the autophagy induction stage but blocked autophagy maturation processes resulting in a decrease of autophagy flux. Normal lysosome function is critical for autophagy maturation; thus, we then explored the effects of SF on lysosomes by examining the levels of LAMP1/2 and TFEB. As shown in Figure 1C,D, SF significantly reduced LAMP2 levels (0.50 ± 0.03 fold, *p* < 0.05) but not LAMP1 levels (*p* > 0.05) compared to sham. Interestingly, we also found that SF significantly increased TFEB levels (1.80 ± 0.25 fold, *p* < 0.05). Taken together, these data indicate that short-term SF can dysregulate mechanisms underlying lysosomal maturation which affected lysosomal degradation and blocked autophagy in the striatum.

### 3.2. Short-Term SF Dysregulated Autophagy in the Hippocampus

**Figure 2:**
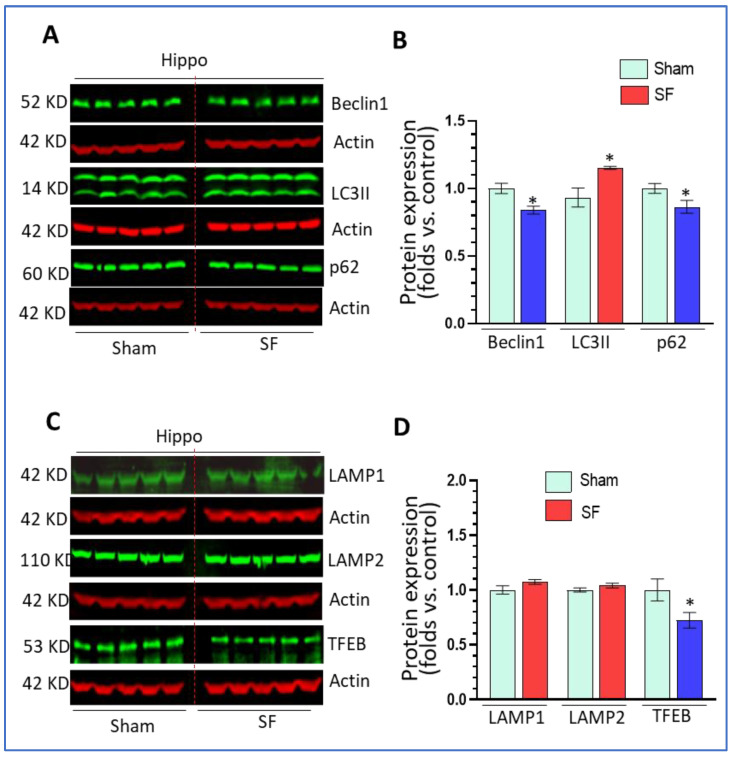
Short-term SF dysregulated autophagy in the hippocampus. (**A**,**B**). Beclin1, and p62 Scheme 3. II significantly increased its levels in the striatum of SF-experienced mouse brains compared to the sham controls (* *p* < 0.05, n = 5). (**C**,**D**) TFEB significantly decreased its levels in the striatum of SF-experienced mouse brains compared to the sham controls (* *p* < 0.05, n = 5). LAMP1 and LAMP2 did not show significant changes in their levels between the SF and sham groups (* *p* > 0.05, n = 5). For all Western blots, β-actin were served as a protein load control. Data were expressed as means ± SEM and were analyzed using Student’s *t*-tests (* *p* < 0.05 versus sham). (Original Western Blot Figure see Supplementary Figure S2).

We next investigated the status of autophagy processes in the hippocampus in both SF- and sham- experienced mice. Surprisingly, we found that 5-days of SF decreased beclin1 levels (0.84 ± 0.03 fold, *p* < 0.05), increased LC3II levels (1.21 ± 0.01 fold, *p* < 0.05) and decreased p62 levels (0.86 ± 0.05 fold, *p* < 0.05) (Figure 2A,B) compared to sham. Short-term SF did not change the levels of LAMP1 and LAMP2, but significantly decreased TFEB levels (0.72 ± 0.07 fold, *p* < 0.05) (Figure 2C,D). The effects of SF on the levels of beclin1, p62, and TFEB in the hippocampus were opposite to the effects observed in the striatum. The upregulation of LC3II and decrease of p62 levels suggested that short-term SF increased autophagy flux in the hippocampus.

### 3.3. Short-Term SF Had No Impact on Autophagy in the Frontal Cortex

**Figure 3:**
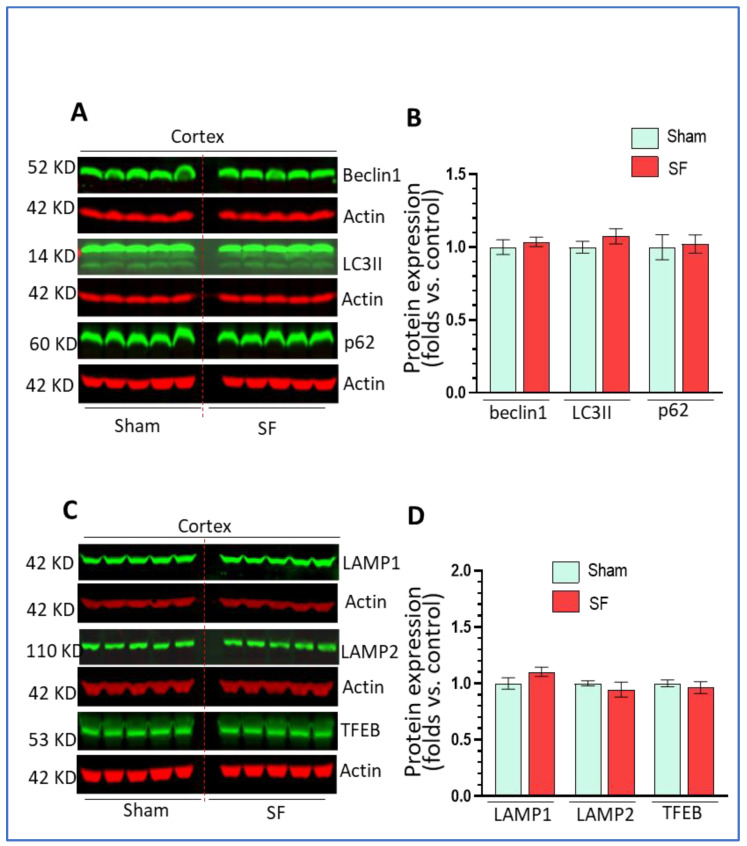
Short-term SF had no impact on autophagy in the frontal cortex. (**A**,**B**) Beclin1, LC3II, and p62 did not change their levels in the striatum of SF-experienced mouse brains compared to the sham controls (* *p* > 0.05, n = 5). (**C**,**D**) LAMP1, LAMP2, and TFEB did not change their levels in the striatum of SF-experienced mouse brains compared to the Scheme 0. n = 5). For all Western blots, β-actin were served as a protein load control. Data were expressed as means ± SEM and were analyzed using Student’s *t*-tests (* *p* < 0.05 versus sham).). (Original Western Blot Figure see Supplementary Figure S3).

Activity in the frontal cortex can reflect sleep intensity and it is vulnerable to sleep disturbances [38,39]. We also explored autophagy processes in this brain region. Similarly, we examined the levels of beclin1, LC3II, p62 as well as the levels of LAMP1/2 and TFEB. As shown in Figure 3A–D, we did not find significant changes in their expression levels in the frontal cortex in comparisons between SF- and sham-experienced brains. These results indicated that short-term SF had no significant effects on autophagy in this region which is different from effects reported for chronic SF.

### 3.4. Short-Term SF Activated Microglia in a Region-Specific Manner In Vivo

**Figure 4:**
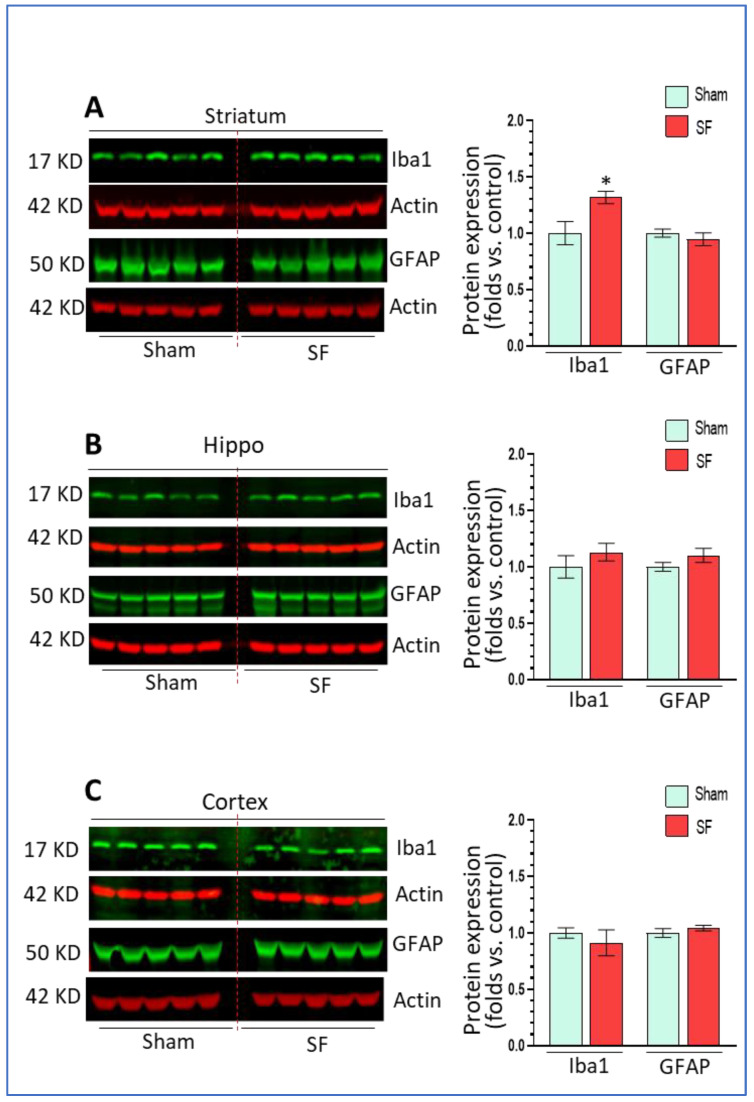
Short-term SF activated microglia in a region-specific manner in vivo. (**A**) Iba1 but not GFAP significantly increased its levels in the striatum of SF-experienced mouse brains compared to sham controls (* *p* < 0.05, n = 5). (**B**) Iba1 and GFAP did not significantly increase their levels in Table 0. n = 5). (**C**) Iba1 and GFAP did not significantly increase their levels in the frontal cortex of SF-experienced mouse brains compared to sham controls. For all Western blots, β-actin were served as a protein load control. Data were expressed as means ± SEM and were analyzed using Student’s *t*-tests (* *p* < 0.05 versus sham). (Original Western Blot Figure see Supplementary Figure S4).

Long-term SF activated microglia in the frontal cortex and HPC in association with autophagy dysregulation, therefore, we assessed whether short-term SF also affected microglia and astrocytes functional status in the brain. In the striatum, SF significantly increased Iba1 levels (1.32 ± 0.05 fold, *p* < 0.05) but did not affect GFAP expression (0.94 ± 0.06 fold, *p* > 0.05) (Figure 4A) compared to sham. In the hippocampus, we did not find any significant changes on the levels of Iba1 or GFAP (1.13 ± 0.07 fold, *p* > 0.05; 1.10 ± 0.06 fold, *p* > 0.05) (Figure 4B). In the frontal cortex, there was no significant changes in either Iba1 or GFAP levels although we observed a trend toward down-regulation in Iba1 levels (*p* > 0.05) (Figure 4C). Taken together, short-term SF activated microglia in the striatum but not in the hippocampus or frontal cortex indicating that there were increased neuroinflammation levels in the striatum.

### 3.5. The Effects of Short-Term SF on Corticotropin Releasing Factor (CRF) Signaling in Extrahypothalamic Regions

**Figure 5:**
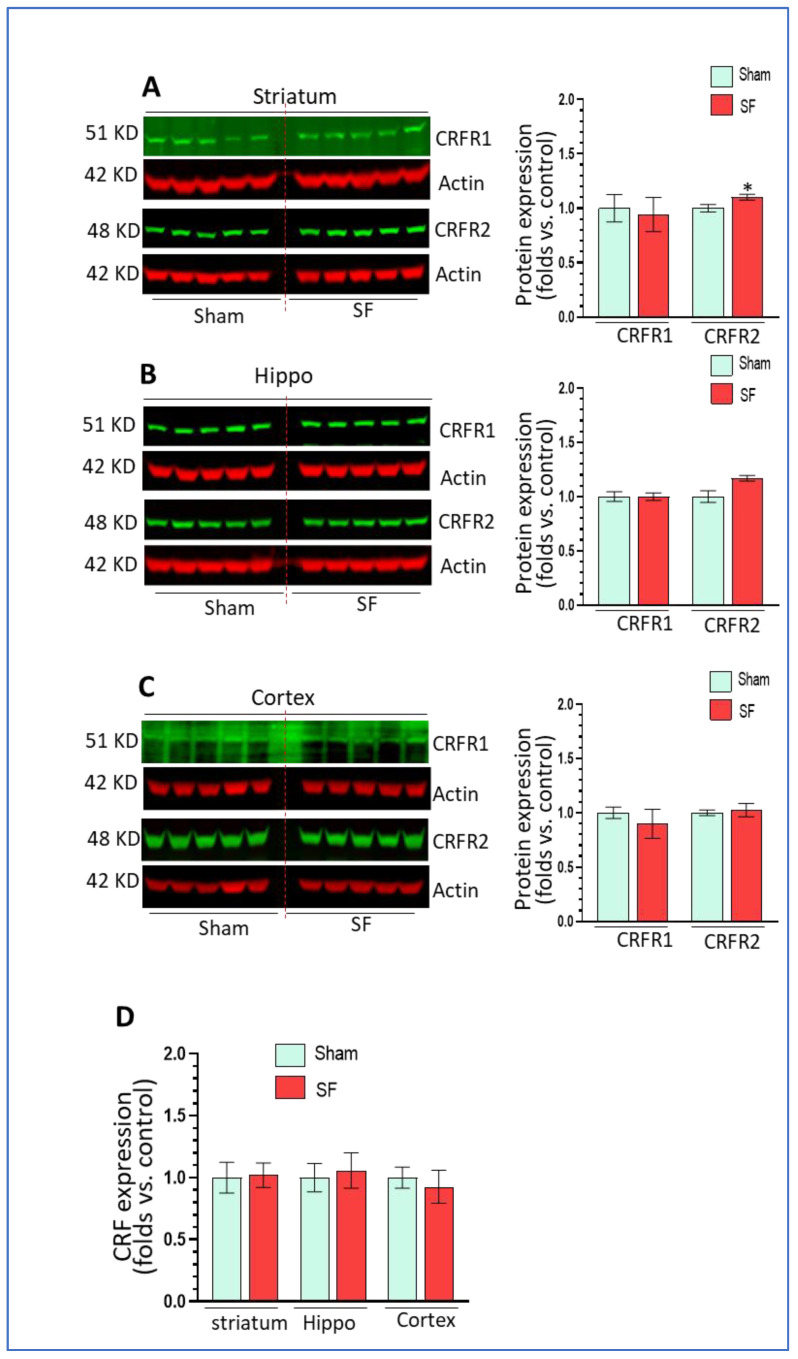
The effects of short-term SF on CRF signaling in extrahypothalamic regions. (**A**) CRFR2 but not CRFR1 significantly increased its levels in the striatum of SF-experienced mouse brains compared to sham controls (* *p* < 0.05, n = 5). (**B**) CRFR1 and CRFR2 did not significantly increase their levels in the hippocampus of SF-experienced mouse brains compared to sham controls (*p* > 0.05, n = 5). (**C**) CRFR1 and CRFR2 did not significantly increase their levels in the frontal cortex of SF-experienced mouse brains compared to sham controls. (**D**) CRF did not significantly increased its mRNA levels in the striatum, hippocampus, or frontal cortex of SF-experienced mouse brains compared to sham controls. For all Western blots, β-actin were served as a protein load control. For all quantitative polymerase chain reactions (qRT-PCR), GAPDH were served as an internal control for quantification. Data were expressed as means ± SEM and were analyzed using Student’s *t*-tests (* *p* < 0.05 versus sham). (Original Western Blot Figure see Supplementary Figure S5).

Sleep disorders are known to be associated with dysregulation of the hypothalamic–pituitary–adrenal (HPA) axis and increased levels of CRF in the brain [27,28,29]. CRF and its cognate receptors CRFR1/2 are widely distributed in extrahypothalamic regions such as the amygdala, hippocampus, and frontal cortex [40] where CRF acts as a neuroregulatory factor for behavioral responses to stress. Here, we explored the effects of SF on CRF signaling in these extrahypothalamic brain regions. We checked the mRNA levels of CRF and the protein levels of CRFR1 and CRFR2. As shown, short-term SF did not increase CRFR1 levels but slightly and significantly increased CRFR2 levels in the striatum (*p* < 0.05, Figure 5A) compared to sham. In the hippocampus, there were no changes in CRFR1 levels and a trend toward upregulation of CRFR2 levels did not reach significance *(p* > 0.05, Figure 5B). In the frontal cortex, we did not observe significant changes in either CRFR1 or CRFR2 levels (*p* > 0.05, Figure 5C). For the mRNA levels of CRF, we did not find any significant changes in any of these three brain regions (*p* > 0.05, Figure 5D). In summary, short-term SF did not strikingly alter CRF signaling in extrahypothalamic bran regions except for an upregulation of CRFR2 in the striatum.

## 4. Discussion

In this study, we explored the effects of short-term sleep disturbance on autophagy, functional status of microglia and astrocytes, and CRF signaling in the brain. Our findings showed that short-term SF dysregulated autophagy in the striatum and hippocampus, activated microglia and increased CRFR2 levels in the striatum but not in the hippocampus or frontal cortex. Short-term SF had no significant impact on astrocyte status or CRF mRNA levels in any of the examined brain regions. In contrast to chronic SF that produces significant microglial activation and autophagy dysregulation in the hippocampus and frontal cortex [36], short-term SF mainly had effects in the striatum. These results suggest that the striatum may be more vulnerable, compared to the hippocampus and frontal cortex, to the effects of short-term sleep disturbance.

Sleep disorders are highly prevalent in Western countries, partially arising from social factors including competition, stress, pressure, and childhood neglect [41]. Nearly 25% of adults in the United States have insomnia and other sleep disorders [42] and abnormal sleep has been identified as a major risk factor contributing to multiple neuroinflammatory and neurodegenerative diseases including PD, AD, and neuropsychiatric diseases [43,44]. The underlying mechanisms responsible for such phenomenon remain elusive, however, accumulating evidence indicates that sleep disturbances can activate microglia and that the resulting increased neuroinflammation levels can lead to synaptic loss and neuronal dysfunction. Previous studies showed that sleep deprivation for 48 h in rats increased the levels of IL1β, TNFα, and IL6 and decreased anti-inflammatory factors IL4 and IL10 in the hippocampus, and led to spatial memory impairment [45] whereas microglia inhibition mitigated spatial memory loss [23]. Sleep restriction (20 h for consecutive 10 days) increased microglial Iba1 levels in the hippocampus in C57BL/6 mice [46]. Chronically sleep-restricted mice and rats also showed similar changes in neuroimmune signaling and neuronal dysfunction [47,48].

While previous studies have identified neuroinflammation as a likely mediator for sleep disorder-induced brain dysfunction, they also have limitations: (1) many investigations employed sleep deprivation which does not model sleep problems commonly observed in human (fragmented sleep); (2) focus on the effects of chronic sleep disturbance on microglia and autophagy dysregulation does not enable fully determining whether pathological changes were causative or outcomes of brain dysfunction; (3) previous studies mainly focused on brain regions (e.g., hippocampus and cortex) which are critical for memory and spatial recognition whereas other regions such as the striatum, which are also critical for regulating sleep activity [26], have seldom been investigated. Therefore, we a used a SF procedure to model sleep disturbance which could better mimic poor sleep quality and interrupted sleep–wake cycles in human. We also employed a 5-day SF protocol in an attempt to determine whether autophagy dysregulation and microglial activation could be initiated with relatively brief sleep disturbances.

Previous studies have indicated that sleep disturbances can regulate autophagy in multiple organ systems in rodents. For example, autophagy was triggered by oxidative stress in the liver of sleep-deprived rats [49]. Paradoxical sleep deprivation induced biological responses in rat masticatory muscles were likely mediated through inflammation and autophagy [50] and abnormal autophagy was linked to paradoxical sleep loss-associated neurodegenerative and patho-physio-behavioral changes in the brain [51]. Sleep deprivation also induced aberrant autophagy in hippocampus neurons [24,25] and inhibition of autophagy in hippocampus improved sleep deprivation-induced cognitive impairment [52]. More relevant to our studies, chronic SF (two months) was capable of dysregulating the endosome-autophagosome-lysosome pathway and microglia-mediated neuroinflammation in the hippocampus and cortex [36]. Here, in agreement with previous findings, we demonstrate that short-term SF also dysregulated autophagy in the striatum and hippocampus. Our findings are novel in two regards: (1) autophagy dysregulation and microglial activation in the striatum were early events induced by SF. Previous investigations consistently showed that chronic sleep disturbances led to increased microglial activation as well as to cognitive and spatial memory impairment (neuronal dysfunction) and that microglial inhibition mitigate these effects. These results suggest that autophagy dysregulation might be responsible for the brain dysfunctions; however, direct evidence supporting a causative role is minimal. Our results showing that autophagy dysregulation in the striatum and hippocampus after 5 days of SF in mice strongly support the argument that autophagy alterations were probably the primary initiator of the increased neuroinflammation and neuronal dysfunction found in the context of chronic sleep disturbances; (2) autophagy dysregulation and microglia activation were mainly found in the striatum after short-term SF (region specific). These findings may provide a potential explanation for the high co-morbidity of sleep disorders and drug addiction. It is known that abused drugs and sleep disturbance affect almost the same neurocircuitry including the striatum and they could accelerate and exacerbate their individual disease courses through this circuitry [26]. Rodent models have revealed that sleep deprivation potentiates cocaine-induced hyper-locomotion and enhances cocaine conditioned place preference [53,54], but the neurological mechanisms underlying this phenomenon remain elusive. Recently, accumulating evidence indicate the involvement of neuroimmune signaling in drug addiction, arguing that elevated levels of neuroinflammation can increase the reward and reinforcing effects induced by abused drugs, thereby leading to enhanced risk for drug addiction [55,56]. Additionally, microglia activation in the ventral striatum promotes cocaine self-administration in mice [57,58]. Therefore, increased microglial activation in reward circuitry might serve as a bridge linking sleep disturbances and drug addiction. This hypothesis is worthy of more investigation.

We did not observe astrocyte activation in the brains of short-term SF mice. However, astrocytes play critical roles in regulating sleep activity. Astroglial calcium activity changes dynamically across vigilance states which is proportional to sleep need and regulation of the sleep homeostat [59,60]. Chronic sleep deprivation (CSD, 18 h for 21 days) increased both Iba1 and GFAP levels in the hippocampus and in the piriform cortex in Wistar rats [48]. Also, CSD increased NLRP3 inflammasome signaling in astrocytes in vivo [61]. Sleep loss promoted astrocytic phagocytosis in the mouse cerebral cortex [62]. CSD induces also proteomic changes in astrocytes of the rat hypothalamus implying that the astrocytes were activated [63]. The discrepancy between our findings and previous studies may be attributed to the different protocols used that differed in the intensity and the duration for sleep disturbance, as well as the animal species (mouse vs. rat). In the brain, microglia are the sensor and initiators of the immune responses, which in turn, activate astrocytes to amplify existing neuroimmune responses [64]. In the earlier response phase of SF, astrocytes may not be activated but would be activated in the context of chronic SF. Our mice were also sacrificed after a 12 h or greater period where they were not disturbed, which potentially could have allowed some normalization to occur.

To identify the possible pathways underlying short-term SF-mediated autophagy dysregulation, we focused on the status of CRF signaling. The CRF/CRFR axis has been shown to dysregulate autophagy in vitro [65,66]. Increased expression of CRF and CRFRs has been found in dorsal striatum, hippocampus, and prefrontal cortex in rats with nicotine or methamphetamine injection, implying that extrahypothalamic CRF signaling was also sensitive to different challenges [67,68]. However, we did not observe significant changes in CRF mRNA levels in the three brain regions that we examined. For CRF receptors, there were no significant effects on their protein levels except the upregulation on CRFR2 in the striatum. It seems that short-term SF had no profound effects on CRF signaling in extrahypothalamic regions in our model. However, we still could not exclude the possibility of the involvement of CRF signaling in striatal microglia activation. It was possible that short-term SF could increase CRF protein levels without altering its mRNA levels or, possibly, short-term SF could have increased CRF production in the hypothalamus which was then exported to the striatum. In supporting this possibility, there exist ample CRF-expressing neuronal inputs to the nucleus accumbens from stress-related brain regions including the paraventricular nucleus of the thalamus, bed nucleus of stria terminalis, etc. [69]. Also, as indicated above, the 12 h or greater period between the end of SF and sacrifice could have allowed time for any upregulation of CRF to normalize.

## 5. Conclusions

We demonstrate that short-term SF dysregulates autophagy in the striatum and hippocampus and increased microglial activation in the striatum. These findings suggest that dysregulated autophagy might be one of the first changes that can herald the substantial neuroinflammation and brain dysfunction that occur in the context of chronic sleep disorders. More investigations are needed to confirm the roles of autophagy dysregulation in neurological symptoms associated with sleep disorders.

## Data Availability

Not applicable.

## Supplementary Materials

The following are available online at https://www.mdpi.com/article/10.3390/life11101098/s1, Figure S1: Short-term sleep fragmentation (SF) dysregulated autophagy in the striatum; Figure S2: Short-term SF dysregulated autophagy in the hippocampus; Figure S3: Short-term SF had no impact on autophagy in the frontal cortex; Figure S4: Short-term SF activated microglia in a region-specific manner in vivo; Figure S5: The effects of short-term SF on CRF signaling in extrahypothalamic regions.
