# Supplementary material for: Short-Term Sleep Fragmentation Dysregulates Autophagy in a Brain Region-Specific Manner
Source: Life (Basel). 2021 Oct 16;11(10):1098. doi: 10.3390/life11101098 (PMC8538758; doi:10.3390/life11101098)
Supplement: Supplementary file 1 [file life-11-01098-s001.zip › life-1419966-supplementary.pdf]

life

MDPI

Article

Short-Term Sleep Fragmentation Dysregulates Autophagy in a Brain Region-Specific Manner

Yan Cheng [1,2,3], Woong-Ki Kim [1,4], Laurie L. Wellman [1,2], Larry D. Sanford [1,2], Ming-Lei Guo [1,3,*]

1 Center for Integrative Neuroscience and Inflammatory Diseases, Eastern Virginia Medical School, Norfolk, VA 23507, USA; chengy@evms.edu (Y.C.); kimw@evms.edu (W.-K.K.); WellmaLL@evms.edu (L.L.W.); SanforLD@evms.edu (L.D.S.)
2 Sleep Research Laboratory, Department of Pathology and Anatomy, Eastern Virginia Medical School, Norfolk, VA 23507, USA
3 Drug Addiction Laboratory, Department Pathology and Anatomy, Eastern Virginia Medical School, Norfolk, VA 23507, USA
4 Department of Microbiology and Molecular Cell Biology, Eastern Virginia Medical School, Norfolk, VA 23507, USA
* Correspondence: GuoM@evms.edu; Tel: +1-757-446-5891

Citation: Cheng, Y.; Kim, W.-K.; Wellman, L.L.; Sanford, L.D.; Guo, M.-L. Short-Term Sleep Fragmentation Dysregulates Autophagy in a Brain Region-Specific Manner. *Life* **2021**, *11*, 1098. https://doi.org/10.3390/life11101098

Academic Editor: Marina Saresella

Received: 28 September 2021
Accepted: 14 October 2021
Published: 16 October 2021

Publisher's Note: MDPI stays neutral with regard to jurisdictional claims in published maps and institutional affiliations.

Copyright: © 2021 by the authors. Licensee MDPI, Basel, Switzerland. This article is an open access article distributed under the terms and conditions of the Creative Commons Attribution (CC BY) license (http://creativecommons.org/licenses/by/4.0/).

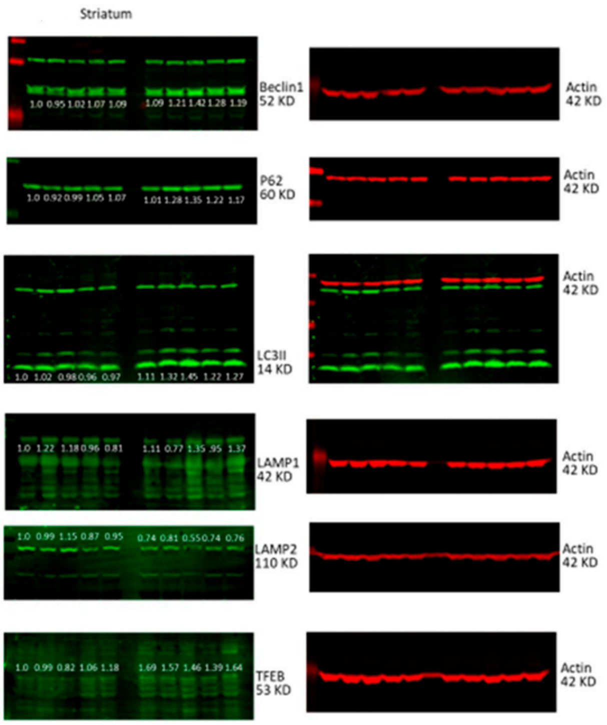

Figure S1. Short-term sleep fragmentation (SF) dysregulated autophagy in the striatum.

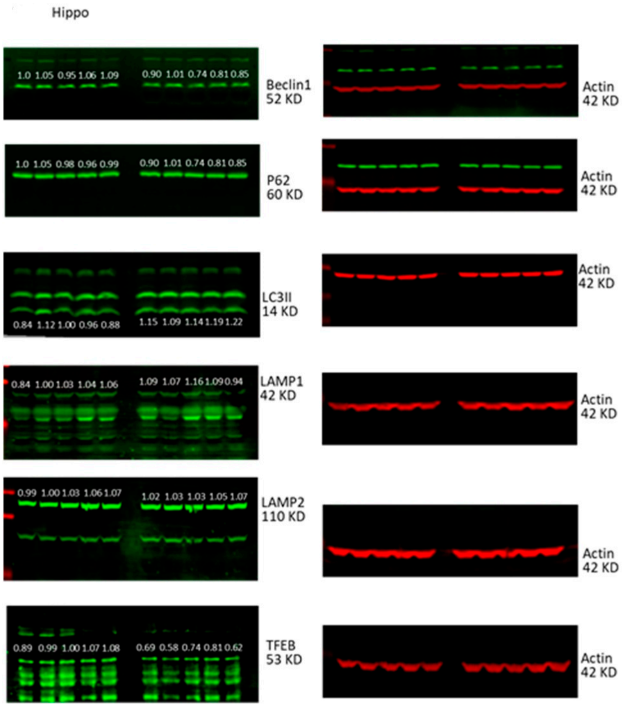

Figure S2. Short-term SF dysregulated autophagy in the hippocampus.

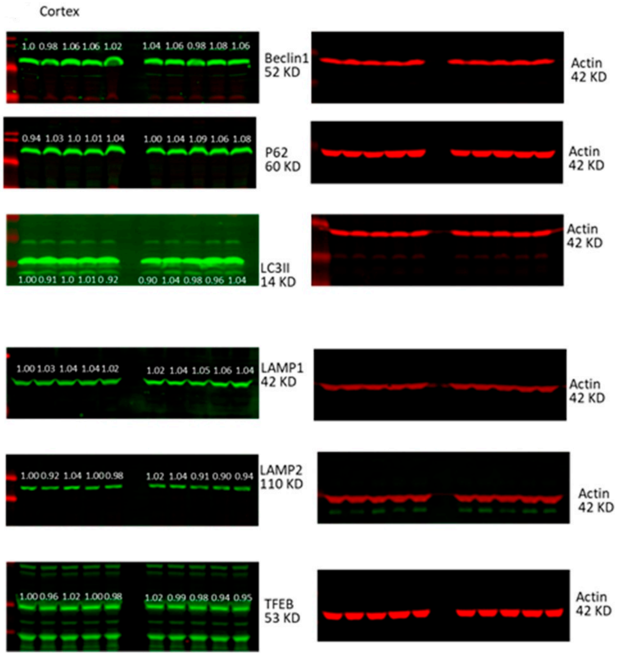

Figure S3. Short-term SF had no impact on autophagy in the frontal cortex.

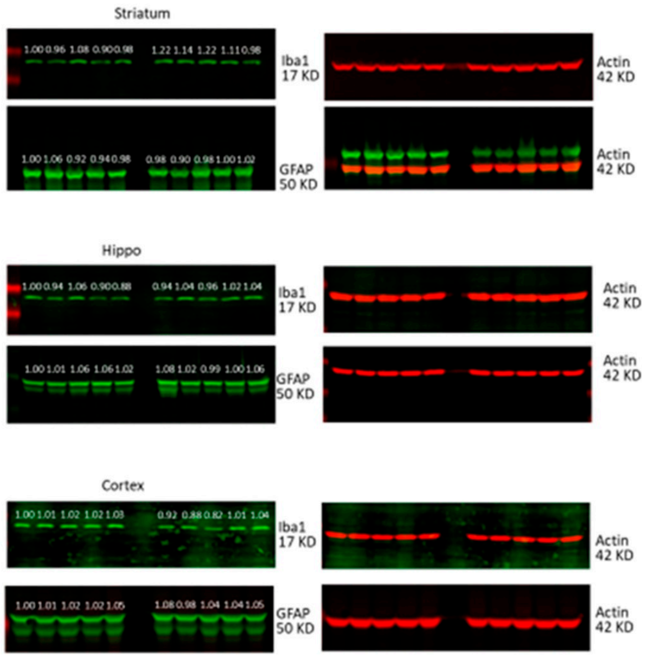

Figure S4. Short-term SF activated microglia in a region-specific manner in vivo

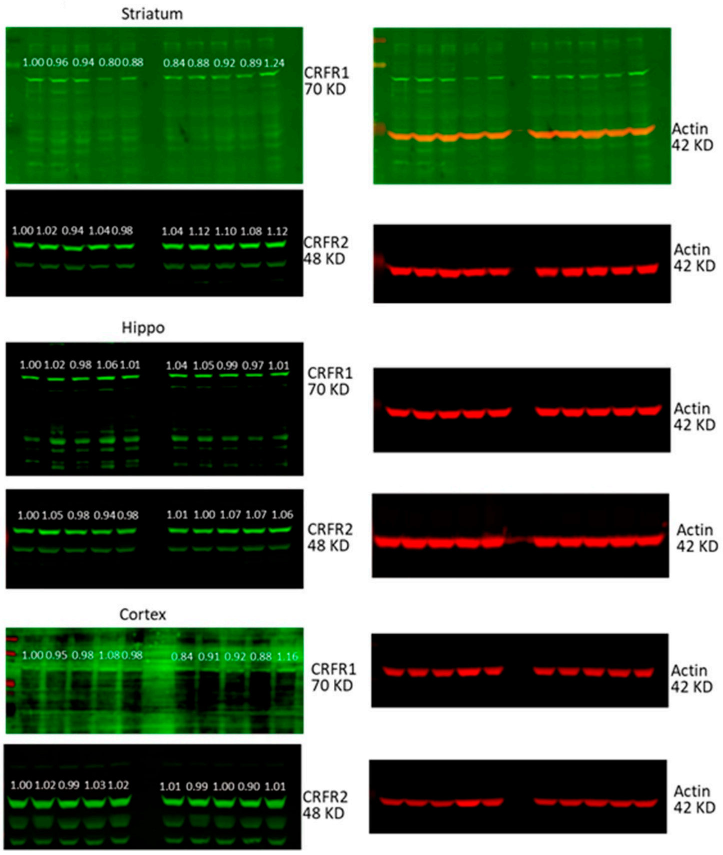

Figure S5. The effects of short-term SF on CRF signaling in extrahypothalamic regions.
